# The Effect of Mild Cyclic Loads on the Stress State of Degenerative Knee Joint Cartilages: A Numerical Study Aided by Experimental Data

**DOI:** 10.3390/biomedicines13123097

**Published:** 2025-12-16

**Authors:** Oleg Ardatov, Vaiga Zemaitiene, Eiva Bernotiene, Arturas Kilikevicius

**Affiliations:** 1 Faculty of Mechanics, Vilnius Gediminas Technical University, LT-10223 Vilnius, Lithuania; 2 Faculty of Medicine, Vilnius University, LT-03101 Vilnius, Lithuania; 3 Department of Regenerative Medicine, State Research Institute Centre for Innovative Medicine, LT-10221 Vilnius, Lithuania; 4 Faculty of Fundamental Sciences, Vilnius Gediminas Technical University, LT-08406 Vilnius, Lithuania

**Keywords:** cartilage, experimental data, finite element method, knee joint, meniscus, numerical modeling

## Abstract

**Background/Objectives**: This study investigates the effect of mild cyclic loads on the stress state of degenerative knee joint cartilages using a combination of experimental data and numerical modeling. **Methods**: A three-dimensional finite element model of the knee joint was developed based on CT scans, incorporating key components such as the femur, tibia, cartilage layers, and meniscus. Special attention was given to the mechanical properties of cartilages, which were determined through high-sensitivity dynamometer tests of cartilage samples. The experimentally obtained force–displacement curves for cartilage samples affected by third-degree gonarthrosis were integrated into the numerical model. This allowed for an in-depth investigation of the interactions between neighboring tissues of the knee joint under cyclic loading and unloading conditions. **Results**: Experimental data revealed nonlinear mechanical behavior of cartilage under loading and unloading conditions, characterized by an elastic hysteresis loop. Experimental results demonstrated that degenerated cartilage, under small stresses (up to 0.13 MPa), retains an elastic hysteresis behavior. The numerical simulation provided insights into the stress distribution within the knee joint components, revealing that even in cases of cartilage degeneration, as long as its structural integrity is maintained, mild loads do not cause sufficient stress concentrators, while the longitudinal tears in the same conditions cause the increment of stress values up to 20%. **Conclusions**: Findings contribute to a better understanding of the mechanical response of degenerative cartilage and offer valuable guidance for the development of therapeutic and rehabilitation strategies for patients with degenerative tissue diseases.

## 1. Introduction

Physiologically, cartilage tissue requires constant mechanical loading to maintain cartilage homeostasis [1]. However, excessive mechanical loading (overloading) leads to degenerative changes in the cartilage and, as the damage progresses, to changes in other surrounding tissues of the joint. This results in degenerative joint diseases such as osteoarthritis (OA), which is associated with tissue inflammation, pain, stiffness, and limitation of movement due to joint degeneration, and which significantly impairs the quality of the patient’s life [2]. OA is treated with intra-articular injections, of which the most commonly used and whose mechanisms of action have been validated in efficacy studies in the treatment of OA include corticosteroid injections, which reduce inflammation by inhibiting the production of inflammatory cytokines such as interleukin-1 (IL-1), leukotrienes, prostaglandins and metalloproteinases [3], hyaluronic acid injections, which improve the viscoelastic properties of synovial fluid [4], Platelet-Rich Plasma (PRP), which was shown to promote tissue regeneration and reduce inflammation. The remaining options include painkillers or non-medication orthopedic measures such as implants, physiotherapy, etc. [5]. The effect of physical therapy on joint tissues in degenerative joint disease, which focuses on the mechanical loading of joint tissues during exercise to ensure cartilage homeostasis, nutrition, and metabolism, is increasingly acknowledged as one of the potential solutions. This is recommended by OARSI as an important strategy for the treatment of OA and is currently used as an alternative to surgical and pharmacological treatments [6,7].

The amount and intensity of mechanical loading induced by physical therapy are very important factors in the condition of joint tissues [1]. Studies to date have shown a beneficial effect of mechanical loading on the regenerative processes of cartilage damaged by degenerative diseases, but it remains a great challenge to determine which loading parameters are beneficial and which may appear detrimental to the joint cartilage [8].

Light mechanical loading, such as light exercise including walking, swimming, yoga, etc., has been shown to have a beneficial effect for patients with degenerative joint disease [9]. Effective ways to reduce the symptoms of OA include reducing pain, improving the physical function of the joints by increasing the amplitude of movement (increasing the amplitude of bending and stretching), strengthening the muscles around the joint [9], and reducing risk factors for OA by lowering body weight and thus reducing the effects of excessive mechanical loading on the joints. Light exercise with gentle mechanical loading has been shown to increase the number of chondrocytes in cartilage and promote the synthesis of EMC components (glycosaminoglycan) [8,9], as well as to reduce chondrocyte apoptosis through intracellular signaling pathways. Meanwhile, studies by us and others have shown that overloading promotes the pathogenesis of degenerative diseases and induces lesion progression by decreasing the levels of ECM components, glycosaminoglycans, and proteoglycans in in vitro studies [8], and by increasing cartilage damage in ex vivo animal models [7,10]. However, knowledge on the frequency, duration, and intensity of mechanical loading in in vitro experimental tests on cartilage or cartilage cell samples, as well as in clinical trials of patients exercising under physiological (in vivo) conditions that would induce a beneficial effect on cartilage tissue, and how to optimally maintain the resulting effect, is still limited [11]. The threshold of when mechanical loading is considered mild and when it is considered overload has not yet been precisely defined, with a wide range of loading applied in different experiments, from 0.015 MPa to 3.5 MPa, 0.1 Hz to 1 Hz in frequency, and time intervals from a few minutes a day, up to a few hours a day [2,8], and it is unclear which is the optimal exposure to achieve long-lasting beneficial results. In addition, for each experiment, both in vitro and in vivo, it is necessary to take into account other important factors: the area of the joint most stressed by the mechanical load, the integrity of the tissues, the degree of damage, and the adjacent forces other than the mechanical compressions that are experienced during the mechanical loading, such as creep, stress forces, etc. [2].

Previous studies have shown that the physiological mechanical load applied to the knee joint is in the range of 0.4–2.0 MPa [12]. Numerous studies have been conducted using different in vitro mechanical loading systems, such as commercially available Flexcell [8], which applies dynamic loading based on vacuum compression, or in-house developed systems, such as Tribometer [13]. The latter is a multi-axial loading bioreactor that can simulate the realistic motion experienced by a joint, taking into account the additional forces acting on the joint during movement, such as shear force, etc. [13]. However, there is still no precise limit to the extent of load that ensures the physiological welfare of cartilage, and the threshold of load when it turns into the harmful overload.

Unlike our previous studies [14,15], this research focuses on the effect of mild loads on stressing the model to the point of instability or exceeding the yield strength. For this purpose, we use highly sensitive equipment, such as the SAUTER FH 5 force gauge, (Kern & Sohn GmbH, Balingen, Germany),a to determine the mechanical properties of cartilage samples from differently damaged locations and to identify differences in their stressed state during loading and unloading. The study of the mechanical behavior of knee joint components under low loads may be useful for determining the intensity and frequency of both effective exercise recommended to maintain the health of patients with degenerative tissue diseases and for experimental studies on cartilage tissue samples.

## 2. Materials and Methods

### 2.1. Geometry and Structure of the Model

**Figure 1:**
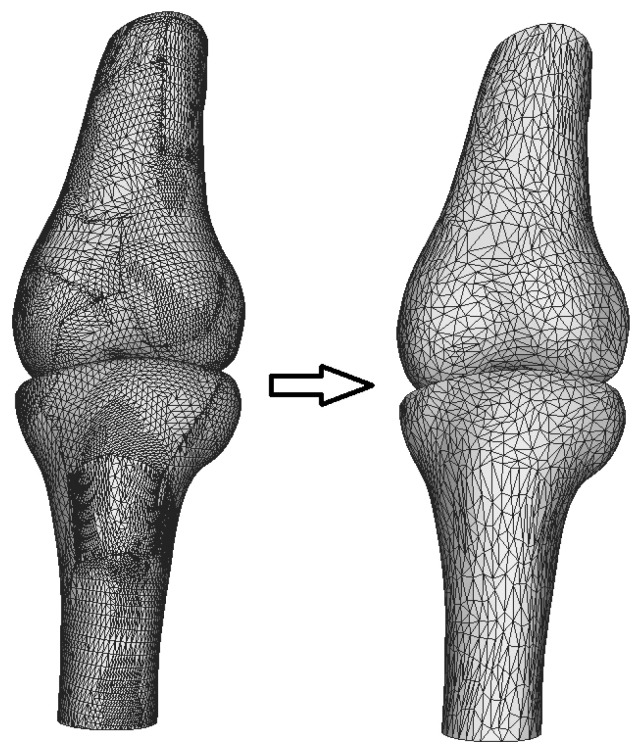
Refinement of initial STL geometry.

The development of the three-dimensional numerical model of the knee joint followed several key steps. First, computed tomography (CT) images were processed using the free open-source software 3D Slicer [16], followed by further refinement in MeshLab [17] to reduce noise and smooth the surfaces. Figure 1 illustrates the enhanced geometry after processing in MeshLab.

As shown in Figure 1, the initial model was simplified by reducing the number of triangles from 53,407 to 15,000, a step taken to optimize computational resources. However, this mesh simplification made the surface angular, so Laplacian smoothing was applied using MeshLab to restore the smoothness lost during the simplification. The output STL file from MeshLab was then imported into SolidWorks [18], where final rendering of the mesh was completed, and the surfaces were converted into solid models of the femur and tibia.

**Figure 2:**
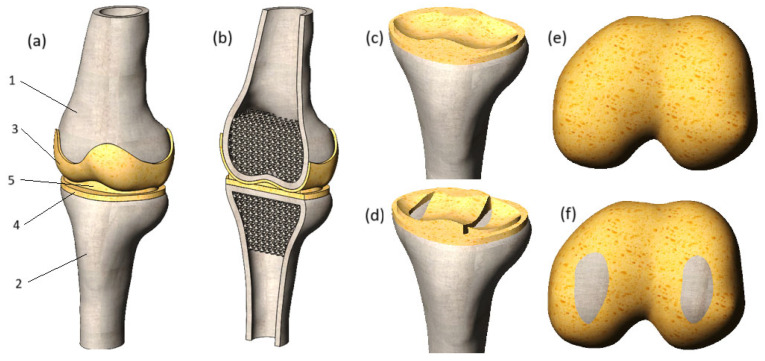
(**a**) Three-dimensional model of the knee joint: 1—femur, 2—tibia, 3—femur cartilage, 4—tibia cartilage, 5—meniscus. (**b**) Model section view; (**c**) tibia cartilage with preserved integrity; (**d**) tibia cartilage with loss of integrity; (**e**) femur cartilage with preserved integrity; (**f**) femur cartilage with loss of integrity.

In this SolidWorks environment, tibia cartilage matching the articular surfaces of the bones was added, with a uniform thickness of 2 mm throughout. In the subsequent stage, the meniscus and femur cartilage were integrated into the model components. The final numerical model is presented in Figure 2.

A section view of the model is shown in Figure 2b. The cortical bones of the tibia and femur were modeled as thin features, with a thickness of 4.5 mm for both bones. The trabecular tissue of the tibia and femur was represented as a porous structure, achieved by applying regular spherical cuts. The diameter of these cuts was set to 6 mm, with the distance between their centers set to 5 mm. As a result, the bone volume to total volume (BV/TV) ratio was calculated to be 0.2, aligning with the BV/TV ratios reported in [19].

To thoroughly investigate the impact of degenerative changes on the stress state of cartilage, this study examines two distinct cases: cartilages with preserved integrity (Figure 2c,e) and damaged cartilages with loss of integrity (Figure 2d,f), which can appear in cases of trauma or long-term effects of degenerative impact. The damages to the tibia cartilage and meniscus were modeled by removing the volume of the cartilage component next to the surface of the bone. This method allowed the reflection of the longitudinal tear of the tissues. As for the femur cartilage, its integrity was compromised by replacing cartilage tissue with bone. As a result, the affected areas of the femur cartilage exhibit increased stiffness, which is uncharacteristic of healthy soft tissues and is likely to negatively impact the mechanical interaction of the joint components.

It is important to highlight that the initial geometry underwent three stages of processing, which introduced distortions to the original shape and volume. Although the model retains the characteristic curvature of the bone, the exact geometric errors remain undeterminable due to the numerous refinements applied to the initial file. Additionally, the model has certain limitations, such as the absence of ligaments, making it suitable only for compression load analysis. Assessments involving rotational or flexional forces may yield unreliable results.

### 2.2. Mechanical Properties of Model Components

The bone (both cortical shell and cancellous tissue) was modeled as a perfectly elastic continuum. The mechanical properties of bone and meniscus were set according to the values offered in [20]. They are presented in Table 1.

**Figure 3:**
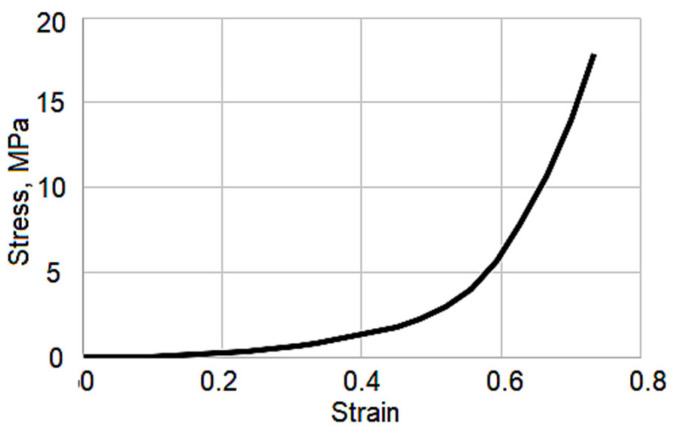
Stress–strain curve illustrating the mechanical properties of the meniscus [14].

The yield stress of the bone was set to 140 MPa [21]. The meniscus was modeled as a perfectly elastic nonlinear material. To determine its mechanical behavior, the stress–strain curve offered in [14] was involved in the study (Figure 3). The presented curve reflects the 3rd stage of osteoarthritis.

### 2.3. Problem Formulation

The mechanical response of the joint components was described using nonlinear elastic theory [18]. Under dynamic loading, the system must satisfy the equilibrium condition at each increment in time, *t* + ∆*t*, given by:

(1) MU″it+∆t+CU′it+∆t+Kit+∆t∆Uit+∆t={R}t+∆t−Fi−1t+∆t,

where [*M*] and [*C*] represent the mass and damping matrices, while *^t^*^+∆*t*^[*K*]^(*i*)^ denotes the stiffness matrix evaluated at iteration *i*. The external load vector *^t^*^+∆*t*^{*R*} and the internal force vector from the previous iteration *^t^*^+∆*t*^{*F*}^(*i*−1)^ enter the equilibrium equation together with the incremental displacement *^t^*^+∆*t*^[∆*U*]^(*i*)^, velocity *^t^*^+∆*t*^{*U*′}^(*i*)^, and acceleration *^t^*^+∆*t*^{*U*″}^(*i*)^. Damping was not included in the analysis, and therefore, the damping matrix was set to zero.

Employing the implicit time integration Newmark–Beta scheme and using Newton’s iterative method, the above equations are expressed in the form:

(2) [K](i) t+∆t∆U(i)={R}(i)t+∆t,

where *^t^*^+∆*t*^{*R*}^(*i*)^ represents the effective load vector, and *^t^*^+∆*t*^[*K*]^(*i*)^ denotes the effective stiffness matrix. To perform calculations, the SolidWorks Simulation module is used.

The applied theory has certain limitations that must be acknowledged. Firstly, this study focuses exclusively on cases of instantaneous loading, excluding the potential long-term consequences of sustained loads. A significant challenge arises in verifying the results due to the inherent variability in tissue properties, making comparisons to a universal standard impractical. Despite this, we aim to capture the general trends in stiffness transfer changes associated with tissue degeneration.

Additionally, it is important to note that the theory of nonlinear elasticity does not account for biological processes occurring within tissues. However, since the components of the knee joint system under consideration are treated as mechanical entities, this theory is deemed suitable for identifying the primary patterns of changes in the model’s stressed state.

To achieve this goal, the von Mises criterion was applied, as defined in Equation (3):

(3) $$\sigma_{y}=\sqrt{\frac{{\left(\sigma_{1}-\sigma_{2}\right)}^{2}+{\left(\sigma_{2}-\sigma_{3}\right)}^{2}+{\left(\sigma_{3}-\sigma_{1}\right)}^{2}}{2}},$$

where *σ*_1_, *σ*_2_, and *σ*_3_ are the maximum, intermediate, and minimum principal stresses, respectively. To assess the strength characteristics of the modeled bone structures, the calculated von Mises stress values were compared to the yield strength specified in Section 2.2.

### 2.4. Clinical Information and Cartilage Explants Preparation Techniques

Human III stage OA knee articular cartilage fragments were obtained from a knee arthroplasty surgery at Vilnius Santara Hospital (Bioethics Committee Permission No. 158200-14-741). Macroscopic assessment showed that the excised joint fragments were clearly damaged, with cartilage wear and cartilaginous tissue up to 0.1 mm thick in places, with almost no underlying bone tissue. The excised cartilage fragments were washed several times in PBS with 2% penicillin and streptomycin (PS) (Thermo Fisher Scientific, Waltham, MA, USA), transferred to a Petri dish, and flat, round explants, 5 mm in diameter and 3 mm height, were excised using a biopsy needle and processed for further analysis.

### 2.5. Experimental Determination of Cartilage Mechanical Properties

**Figure 4:**
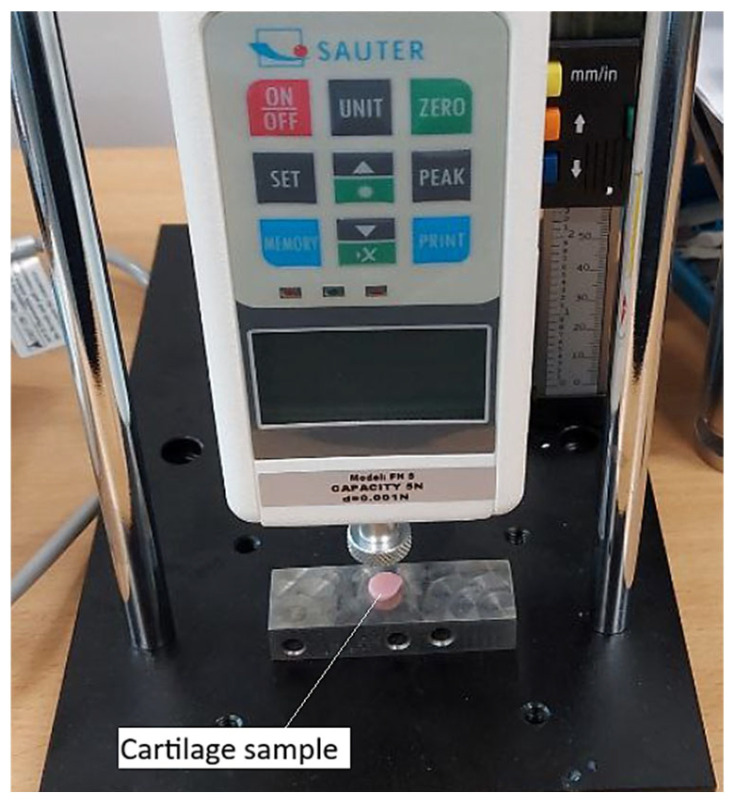
Compression of a cartilage sample using a SAUTER FH 5 force gauge.

To determine the mechanical properties of cartilage affected by third-degree gonarthrosis, the SAUTER FH 5 digital force gauge (Figure 4) was used. Its application was justified by its high precision, supported by technical specifications such as a maximum measurable force of 5 N and a resolution of 0.001 N. These characteristics enabled accurate detection of even the slightest force variations under low-intensity loads.

**Figure 5:**
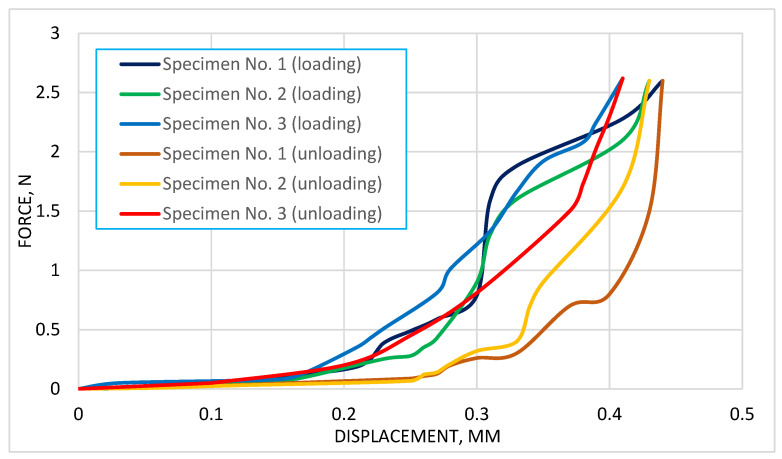
Relation between force and displacement for 3 cartilage specimens.

The high sensitivity of the device made it possible to identify the nonlinear mechanical behavior of cartilage, characterized by differences in responses during loading and unloading phases. An elastic hysteresis specific to degenerative tissue was observed (Figure 5).

As we can see from Figure 5, the graph illustrates the relationship between displacement (mm) and force (N) during the compression of degenerated cartilage specimens using a dynamometer. The data includes both loading and unloading cycles for three specimens. The loading curves for all specimens exhibit a nonlinear behavior, with a gradual increase in force as displacement progresses. In contrast, the unloading curves show a distinct hysteresis loop, which is characteristic of energy dissipation during the loading-unloading cycle.

**Figure 6:**
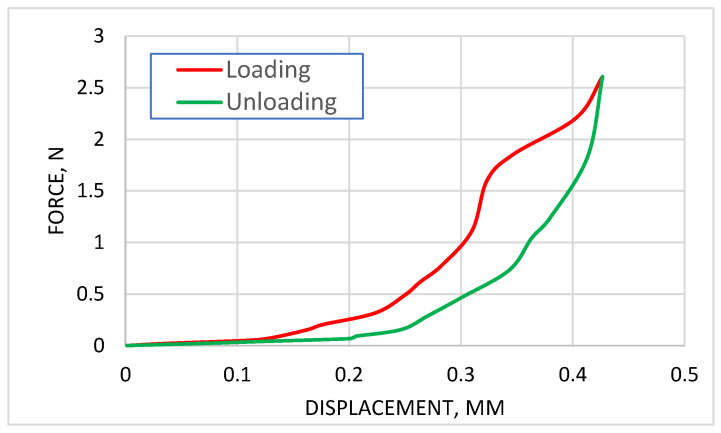
Average displacement–force curve obtained after processing the curves of three specimens.

The average force–displacement curve, derived from previous graph is presented in Figure 6. The graph presents the averaged force–displacement relationship derived from the loading and unloading curves of three degenerated cartilage specimens. The red curve represents the loading phase, while the green curve corresponds to the unloading phase. The averaged loading curve exhibits a characteristic nonlinear behavior, with a gradual increase in force at lower displacements, followed by a steeper rise as displacement progresses. This reflects the progressive stiffening of the cartilage under compression. The unloading curve is distinct from the loading curve, forming a hysteresis loop indicative of energy dissipation during the load-unload cycle. The area enclosed by the loop quantifies this energy loss, which is attributed to the viscoelastic properties and internal friction within the cartilage matrix. The smoothed nature of the averaged curves provides a clearer representation of the general mechanical response of degenerated cartilage to mild compressive loads, capturing both the elastic and energy-dissipative behavior of the tissue.

**Figure 7:**
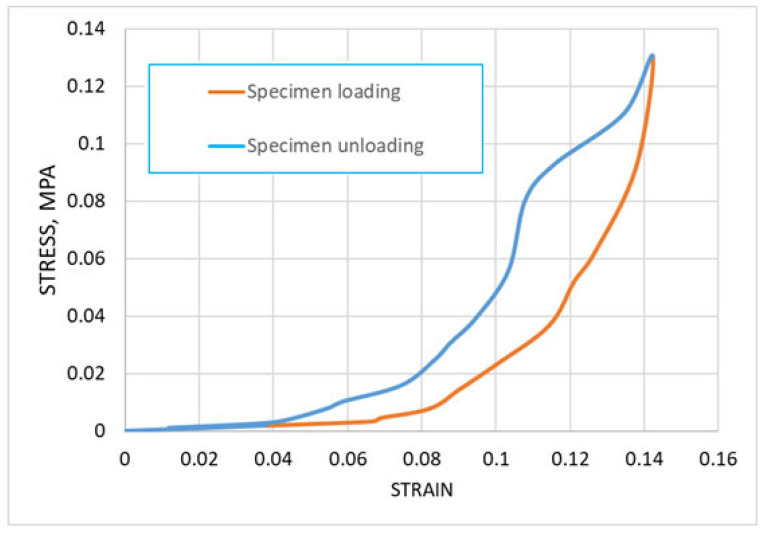
The stress–strain curve obtained from average displacement–force curve.

The stress–strain curve obtained from the average displacement–force curve is presented in Figure 7. The graph illustrates the stress–strain relationship during the compression of degenerated cartilage specimens, with separate curves for the loading (blue) and unloading (orange) phases. The loading curve demonstrates an expected nonlinear increase in stress as strain progresses, with a gradual initial rise followed by a sharper increase at higher strain levels. This indicates the characteristic stiffening behavior of cartilage under compressive deformation. The unloading curve deviates from the loading curve, forming a hysteresis loop that could highlight the viscoelastic nature of the cartilage. The enclosed area between the loading and unloading curves represents the energy dissipated during the loading-unloading cycle. This energy loss is a result of internal friction and structural reorganization within the cartilage matrix. The transition from stress accumulation during loading to stress relaxation during unloading further emphasizes the mechanical and energy-absorbing properties of the tissue under compressive strain. This information can be valuable for understanding the mechanical behavior of degenerated cartilage under physiological loading conditions.

To incorporate the experimentally measured mechanical response of degenerated cartilage into the numerical model, the stress–strain loading and unloading curves were directly implemented in SolidWorks Simulation as tabulated material data. The software interpolates the tabulated points to obtain the tangent stiffness during each iteration, allowing the finite element model to reflect the viscoelastic-like hysteresis behavior observed in the experiments without assuming an a priori constitutive equation.

### 2.6. Loading Scheme and Boundary Conditions

**Figure 8:**
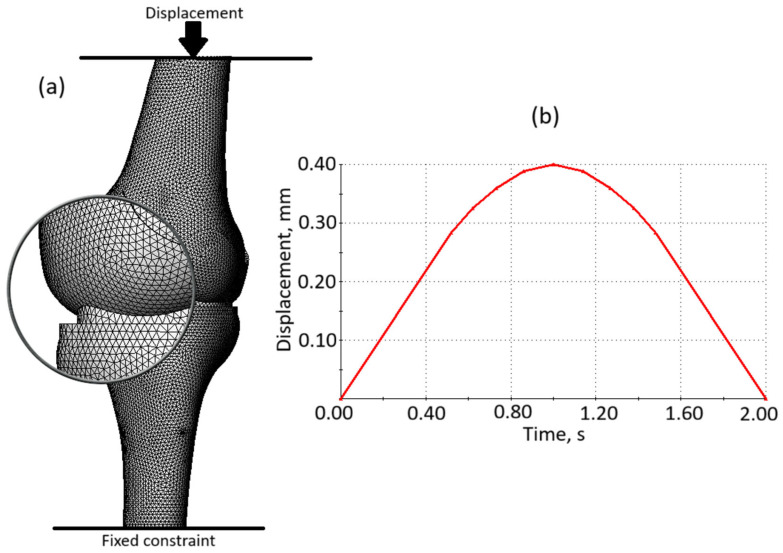
(**a**) The loading scheme of finite element model; (**b**) Loading time curve.

The loading scheme is presented in Figure 8a. As shown in the image, the finite element model is subjected to an external load in the form of displacement applied to the upper surface of the femur. This displacement is directed strictly vertically, inducing compression on the model. The lower surface of the tibia is fixed in place, ensuring that the model remains stationary during the application of the load.

The time curve of loading is shown in Figure 8b. The graph illustrates a loading-unloading cycle, showing the relationship between displacement (measured in millimeters, mm) and time (measured in seconds, s). The curve starts at the origin (0, 0), representing no displacement at the initial time. Description of the cycle: during the loading phase, the displacement increases gradually over time, forming an upward curve. The maximum displacement is equal to 0.40 mm and is reached at 1.00 s. The imposed 0.40 mm compression resulted in reaction forces of approximately 250 N in the model with preserved soft tissue integrity and 270 N in the degenerative model, which indicates low-intensity loading and confirms the absence of overload. The unloading phase starts, respectively, at 1.00 s: the curve follows a similar path downward, indicating a gradual decrease in displacement. At 2.00 s, the displacement returns to 0 mm, completing the loading-unloading cycle.

As we can see from Figure 8a, the model was divided into volumetric finite elements in the form of tetrahedra to ensure that the mesh conforms to the complex anatomical curvature of the knee joint components. This approach allows for accurate representation of the intricate geometries of the femur, tibia, cartilage, and meniscus, enabling precise analysis of stress distribution and mechanical behavior within the joint. Total number of elements—193,998, total number of nodes—310,387. The model is defined by 907,165 degrees of freedom.

The equilibrium equations are solved by applying the Intel Direct Sparse solver.

## 3. Results

**Figure 9:**
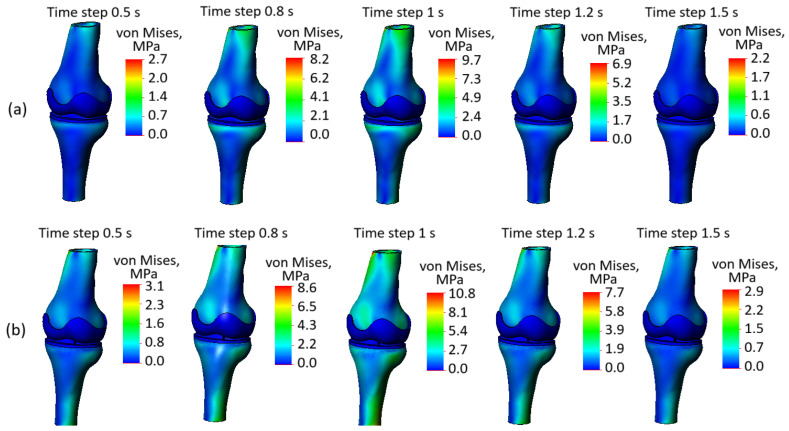
(**a**) Stress state plots of the model with preserved soft tissue integrity across various time steps; (**b**) stress state plots of the model with compromised soft tissue integrity across various time steps.

Stress state plots of the whole knee joint model are presented in Figure 9. As we can see, the stress distribution depends both on the time step, which, in turn, determines the magnitude of the applied displacement, and on the integrity of the soft tissues. Furthermore, the stress values are directly influenced by the loading phase. The obtained plots demonstrate the von Mises stress distribution in the knee joint for two cases: one with intact soft tissues and another with compromised soft tissue integrity. The plots correspond to time steps of 0.5 s, 0.8 s, and 1 s (loading phase), while time steps of 1.2 s and 1.5 s correspond to the unloading phase.

At the loading phase, the stress values increase as expected. At earlier time steps, such as 0.5 s, the stress concentrations are relatively low, whereas loading reaches its peak, results show significantly higher stress levels, indicating the cumulative effect of the applied load. This progression is visible in the increasing number of “brighter” zones representing high-stress regions.

A comparison between the two cases (intact soft tissues and soft tissues with damage) highlights some differences in stress distribution on the bone. In (Figure 9a), where soft tissue integrity is preserved, the stress is more evenly distributed across the joint components. In contrast, in (Figure 9b), where the soft tissue integrity is compromised, there is a noticeable increase in stress concentrations, particularly in the bony components. This indicates that the loss of soft tissue integrity reduces the cushioning effect of the cartilage and meniscus, leading to an uneven load transfer and higher stress levels in the bones.

High-stress regions are observed near the contact areas of the femur and tibia in both cases. However, in (Figure 9b), the magnitude of these stresses is significantly higher due to the impaired load distribution caused by the damaged soft tissues. This stiffer overall behavior in case (Figure 9b) results in the bony components bearing a greater proportion of the applied load.

The color scales further emphasize this difference. In (Figure 9a), the maximum stress values reach 9.7 MPa at certain time steps, while in (Figure 9b), they go up to 10.8 MPa, indicating amplified stress levels when the soft tissues are compromised. This amplification highlights the mechanical challenges introduced by soft tissue degeneration and damage.

Overall, the figure underscores the critical role of soft tissue integrity in maintaining balanced load distribution within the knee joint. When the soft tissues are intact, they act as effective cushions, protecting the bones and reducing stress concentrations. In contrast, compromised soft tissue integrity leads to stress amplification in the bones, increasing the risk of damage and accelerating joint degeneration. These observations are essential for understanding the mechanical consequences of soft tissue degeneration and its impact on joint health, particularly in the context of degenerative diseases.

At the same time it should be noted that the plots presented in Figure 9 are not particularly informative, as they predominantly depict the stress state of the bones, whereas our study focuses on the impact of degenerative changes on soft tissues. Moreover, the observed stress distribution may be misleading, as the red zones noticeable on the upper surface of the femur and the lower surface of the tibia are influenced by Saint-Venant’s principle and are related to the proximity of the applied load and boundary constraints. At the same time, the overall view of the model’s stress state reflects the interaction between rigid and softer tissues. As shown in Figure 9, the stress values within the bone are significantly higher, and the bony components are more prominently “colored”, whereas the cartilage and meniscus remain blue on this plot. This effect is due to the fact that soft tissues deform more easily, leading to much lower stress values.

In the following sections, we will focus specifically on the stress state of soft tissues. This approach will allow for a clearer and more detailed contrast in the plots depicting their stress distribution.

**Figure 10:**
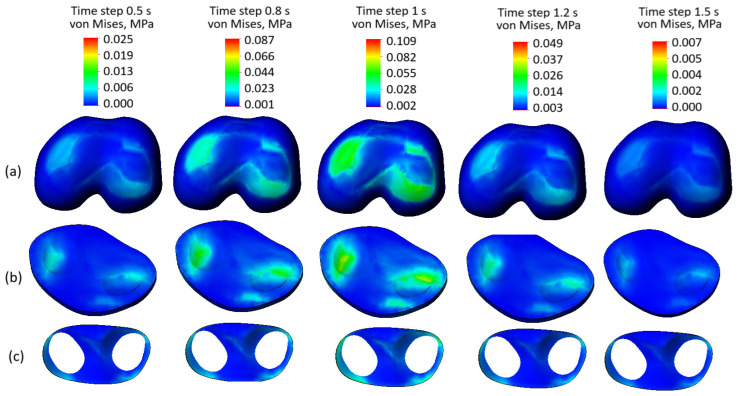
Stress state plots of the model components with preserved soft tissue integrity across various time steps: (**a**) femur cartilage; (**b**) tibia cartilage; (**c**) meniscus.

Figure 10 presents the stress state plots of the model components with preserved soft tissue integrity across various time steps: (Figure 10a) femur cartilage, (Figure 10b) tibia cartilage, and (Figure 10c) meniscus. The time steps range from 0.5 s to 1.5 s, where 0.5 to 1 s corresponds to the loading phase, peaking at 1 s, and 1.2 to 1.5 s represents the unloading phase.

The femur cartilage (Figure 10a) shows a uniform stress distribution throughout all time steps, without any significant stress concentrators, except at time step 0.8 s and the peak at 1 s. During the loading phase (0.5 to 1 s), the stress levels gradually increase, reaching their maximum (0.109 MPa) at 1 s. In the unloading phase (1.2 to 1.5 s), the stress levels decrease from 0.049 MPa to 0.009 MPa, maintaining their uniform distribution.

Similarly, the tibia cartilage (Figure 10b) exhibits a consistent and uniform stress distribution across the mentioned steps. The stress increases gradually during the loading phase, with some localized stress concentrators at the peak of the cycle. During unloading, the stress levels reduce evenly, indicating the tissue’s capability to distribute mechanical loads effectively.

The meniscus (Figure 10c) demonstrates the lowest stress levels among the three components and maintains a uniform stress distribution throughout both loading and unloading phases. No stress concentration is observed in the meniscus, reflecting its effective role in force dissipation within the knee joint. Despite the third-degree osteoarthritis indicated by the curve presented in Figure 3, the uniform stress distribution observed highlights that the preserved integrity of soft tissues remains a crucial factor in load transmission and tissue interaction.

In summary, the stress distributions in all components—femur cartilage, tibia cartilage, and meniscus—are uniform and devoid of any visible stress concentrators across all time steps. This indicates that the preserved soft tissue integrity ensures balanced stress distribution within the knee joint during both loading and unloading phases. These findings emphasize the critical role of intact soft tissues in maintaining the mechanical functionality of the joint.

**Figure 11:**
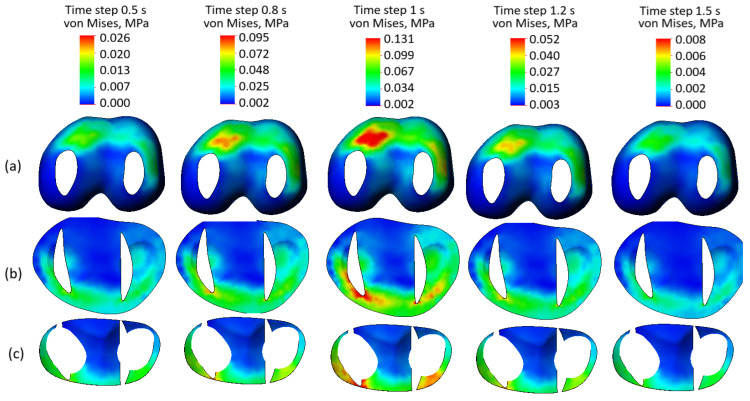
Stress state plots of the model components with damaged soft tissue integrity across various time steps: (**a**) femur cartilage; (**b**) tibia cartilage; (**c**) meniscus.

Stress state plots of the soft knee joint components with damaged tissue integrity across various time steps are presented in Figure 11. Compared to the case with preserved tissue integrity, the stress distribution in the femur cartilage (Figure 11a) shows higher values (0.0131 MPa versus 0.109 MPa at the peak of the cycle) and visible stress concentrators, particularly in regions subjected to direct loading. The loss of soft tissue reduces the efficiency of load redistribution, leading to localized stress peaks and increasing the risk of microdamage. Similarly, the tibia cartilage (Figure 11b) exhibits significantly higher stress levels than in the intact case, with pronounced stress concentration zones. This uneven stress distribution reflects the inability of damaged soft tissues to effectively dissipate loads, resulting in additional strain on the tibial surface. The meniscus (Figure 11c), which typically plays a critical role in load distribution, shows elevated stress values and non-uniform stress patterns. The presence of stress concentrators highlights its diminished capacity to function as a cushioning structure in the joint.

## 4. Discussion

Experimental material properties were obtained from Grade III OA cartilage, meaning that the results of this study primarily describe the mechanical response of tissues at an intermediate stage of degeneration. While the observed trends, such as the absence of stress concentrators under mild loads when structural integrity is preserved, are likely applicable to similar degeneration levels, they should not be directly extrapolated to early-stage (Grade I–II) or severely degraded (Grade IV) cartilage, which may demonstrate different mechanical characteristics.

While the observed stress values remain within non-critical ranges, the emergence of stress concentrators is a concerning factor. These localized high-stress regions are associated with the initiation and propagation of microcracks, which could accelerate cartilage wear and lead to further joint degradation. The inefficient load distribution caused by the loss of soft tissue integrity underscores the mechanical vulnerability of the knee joint under such conditions.

The omission of ligaments and rotational degrees of freedom may influence the absolute stress values reported in this study. Ligaments normally stabilize joint kinematics and constrain excessive translation, and their absence may lead to slightly more uniform load transfer than would occur in vivo. Likewise, suppressing rotational and shear motions reduces the shear component acting on the meniscus, potentially underestimating local stress concentrations, as the meniscus is highly sensitive to shear-induced deformation. Nevertheless, the comparative trends between intact and degenerative soft tissues remain valid, as both models are affected by these simplifications in the same manner.

The performed analysis demonstrates the vital role of soft tissue integrity in ensuring effective load transmission and stress distribution within the knee joint. The findings show that compromised soft tissues not only result in increased stress levels but also lead to the formation of stress concentrators, heightening the risk of microdamage and joint deterioration. These insights emphasize the importance of tailoring treatment strategies, including physiotherapy and other interventions, to the specific condition of the patient’s joint. Proper consideration of the state of the soft tissues is essential to prevent further damage and optimize therapeutic outcomes.

## 5. Conclusions

Using a combination of experimental data and numerical modeling, the research provided the following key findings:

- Elastic Hysteresis in Degenerative Cartilage: Experimental data confirmed the presence of elastic hysteresis in cartilage affected by third-degree gonarthrosis. This behavior, obtained during loading and unloading cycles, reflects the nonlinear nature of the tissue even in the degenerative condition and highlights the importance of investigating cartilage response under varying loads.
- Stress Distribution Insights: Numerical modeling revealed the stress distribution within knee joint components under mild cyclic loads. The analysis showed that degenerative cartilage, as long as its structural integrity remains intact, can sustain small stresses (up to 50 kPa) without inducing significant stress concentrators. This supports the hypothesis that mild loading is beneficial and unlikely to exacerbate cartilage degeneration.
- Therapeutic Potential of Mild Loads: The findings suggest that mild mechanical loads do not harm cartilage with intact structural integrity, even in degenerative states. Instead, they may facilitate joint health by avoiding stress intensification, which is often a precursor to further degeneration.
- Limitations and Future Work: The study highlighted the need for further exploration of loading parameters, such as frequency, duration, and displacement magnitude. Substantially higher displacements or faster cyclic loading could shift the joint response toward conditions approaching mechanical overload, and assessing these effects would help establish an optimal therapeutic regimen. Additionally, the numerical model’s limitations, including the absence of ligaments and rotational forces, indicate avenues for future research.

These results contribute valuable insights into the biomechanical behavior of degenerative cartilage and emphasize the importance of tailoring mechanical loading parameters to preserve joint function and minimize further tissue damage in patients with joint degenerative diseases.

## Figures and Tables

**Table 1:** Mechanical properties of model components.

Model Component	Young’s Modulus, MPa	Poisson’s Ratio
Femur and tibia	7300	0.3
Meniscus	Defined by stress–strain curve (Figure 3)	0.4995
Cartilages	Defined experimentally	0.4995

## Data Availability

The data presented in this study are available on request from the corresponding author.

## Abbreviations

The following abbreviations are used in this manuscript:

BV/TV	Bone volume/total volume
CT	Computed tomography
FEM	Finite element method
IL-1	Interleukin
OARSI	Osteoarthritis Research Society International
OA	Osteoarthritis
PRP	Platelet-rich plasma
